# Heterogeneity in benefit finding among breast cancer patients: a latent profile analysis and influencing factors

**DOI:** 10.3389/fonc.2026.1636972

**Published:** 2026-01-28

**Authors:** Wei Wang, Keying Guo, Weina Du, Ling Cheng, He Gao, Zhongtao Zhou, Jing Zhang

**Affiliations:** 1 College of Nursing, Bengbu Medical University, Bengbu, Anhui, China; 2 College of Mental Health, Bengbu Medical University, Bengbu, Anhui, China

**Keywords:** benefit finding, breast cancer patients, influencing factors, latent profile analysis, machine learning

## Abstract

**Objectives:**

This study investigates factors influencing benefit finding among breast cancer patients based on social cognitive theory and develops a nomogram to predict the probability of low benefit finding in breast cancer patients.

**Methods:**

A study of 666 breast cancer patients in northern Anhui Province (January to December 2024) employed latent profile analysis to identify distinct benefit finding patterns. Potential predictors were identified through univariate analysis, least absolute shrinkage and selection operator regression, and multivariate analysis. Five machine learning algorithms were applied to predict low benefit finding, with performance evaluated via calibration and discriminative power metrics and internally validated using bootstrap resampling.

**Results:**

A two-classification model best fits the data, identifying the low benefit finding category (35%) and the high benefit finding category (65%). XGBoost outperformed other models and was selected as the final model. The model achieved an AUC of 0.945 on the validation set. SHAP analysis quantified each variable’s contribution to predictions, revealing age, medication adherence, anxiety, social support, and depression as key determinants of benefit finding.

**Conclusion:**

This study applied social cognitive theory to examine factors affecting benefit finding in breast cancer patients, focusing on environmental, individual, and behavioral domains. Results showed strong performance by the XGBoost classifier. The developed nomogram aids healthcare providers in swiftly identifying patients with low benefit finding, enabling personalized interventions to mitigate adverse psychological effects and improve long-term outcomes.

## 1 Introduction

Cancer is a major public health challenge, significantly threatening population health and serving as a critical determinant of the global disease burden (1). In 2022, data from the International Agency for Research on Cancer (IARC) indicated that there were almost 20 million new cancer diagnoses worldwide, with cancer fatalities totaling 9.7 million (2). Breast cancer, a major contributor to cancer-related deaths globally, represents a substantial risk to women’s health (3, 4). In 2020, breast cancer emerged as the leading cancer diagnosis globally in women, with around 2.26 million new cases (5). Projections indicate that the global annual incidence of new cancer cases will surpass 3 million in the near future (6). Research shows that breast cancer incidence in China is the highest worldwide, accounting for 17.6% of all cases globally (7). The diagnosis of cancer and the need for invasive treatments often result in negative outcomes for breast cancer patients, leaving them vulnerable to both psychological and physical distress (8). However, studies have demonstrated that such negative impacts can, in some cases, lead to positive life changes (9–12). This favorable change in one’s way of living allows certain individuals to recognize various personal, social, psychological, and spiritual advantages that arise after experiencing traumatic events, a concept known as benefit finding (13, 14).

Benefit finding (BF) refers to individuals’ positive coping strategies in response to traumatic events. It often involves cognitive reappraisal, which helps to elicit positive emotions and promote adaptive behaviors (15). BF encompasses various domains, such as acceptance, familial relationships, worldview, personal growth, social connections, and health (15). Studies have indicated that patients can derive benefit finding from the negative impacts they experience (16, 17). Tennen also believes that benefit finding involves recognizing potential benefits in the face of adversity. This positive transformation can foster psychological growth and promote a healthier lifestyle (18). Furthermore, relevant studies have shown that BF reflects the favorable transformations individuals may undergo post-cancer diagnosis and treatment, fostering improved psychological well-being (19).

Benefit finding is influenced by psychological, environmental, and illness-related factors. Qiu X’s research team selected 319 early-stage cancer patients undergoing treatment as the study participants and conducted a cross-sectional study (20). The research revealed a significant correlation between perceived social support and positive outcomes in benefit finding. Both Lin Y and Lassmann’s research teams have confirmed a significant correlation between anxiety, depression, and benefit finding (18, 21). Cancer staging also influences benefit finding levels (22), with higher stages of cancer associated with lower BF scores. These variables were selected not only for their predictive value, as demonstrated in prior studies, but also to capture a comprehensive understanding of the multifaceted influences on benefit finding among breast cancer patients. Appreciating these influences is essential for improving patients’ prognosis and overall outlook.

Social Cognitive Theory centers on three core components: triadic interaction theory, observational learning, and self-efficacy (23), with triadic interaction theory being the most fundamental. This theory emphasizes that environmental factors, personal factors, and behavioral responses are independent yet mutually influential. Presently, social cognitive theory is extensively utilized within oncology psychology research (24, 25). This study integrates conceptual elements of behavioral responses, individual factors, and environmental factors based on SCT. Using a conceptual framework and literature review, we perform a dynamic evaluation of benefit finding in breast cancer to accurately pinpoint its predictive factors.

BF is a complex process influenced by the interaction of multiple factors. Moreover, BF shows significant individual differences, with varying manifestations and influencing factors across different cancer patients (18, 26). Consequently, there may be potential subgroup differences among cancer patients. Existing studies often employ methods like regression analysis and mediation models, which focus on the average effects or overall associations between variables and are unable to capture the latent subgroup differences within patient populations. Latent Profile Analysis (LPA) can precisely identify these unobserved subgroup characteristics, revealing the”within-group consistency” and “between-group differences” in BF influencing factors across different populations, thus overcoming the limitations of traditional regression models in capturing the “group average effect (27, 28).” This approach offers a novel perspective for understanding individual differences in BF among breast cancer patients and for developing targeted intervention strategies.

Although LPA is highly effective in identifying latent profiles, it lacks predictive capability. Machine learning algorithms, however, can efficiently process complex data structures and learning patterns derived from large datasets, complementing LPA by providing predictive functionality (29). In this study, machine learning was employed to leverage LPA classification outcomes alongside other relevant variables to predict the likelihood of benefit finding. This dual approach, combining LPA and machine learning, delivers a nuanced understanding of benefit finding. Through meticulous patient stratification and data-driven predictive models, it enhances treatment precision, efficiency, and patient quality of life, thereby advancing the development of precision medicine.

The primary objectives of this study are: (1) to identify potential categories of breast cancer patients who may benefit from LPA, (2) to develop and validate machine learning models for predicting benefit finding, and (3) to construct simplified and effective nomograms for predicting the occurrence of benefit finding.

## 2 Methods

### 2.1 Study design and participants

This cross-sectional study, conducted between January 5, 2024, and December 10, 2024, employed a convenience sampling method to select 711 breast cancer patients from two tertiary hospitals in Bengbu City, Anhui Province. After excluding 45 cases due to loss of follow-up, a total of 666 valid cases were included for analysis. Inclusion criteria: (1) Diagnosis of breast cancer confirmed through clinical, radiological, and pathological assessments. (2) Age ≥ 18 years. (3) Written consent was secured, with individuals willingly choosing to take part in the research. (4) No history of other cancers or previous cancer treatments. Exclusion criteria: (1) Presence of other cancers or serious comorbidities. (2) Impaired consciousness, inability to cooperate, or cognitive dysfunction. (3) A history of psychiatric disorders.

The research received approval from Bengbu Medical University’s Ethics Committee (No. 2023-280), with informed consent obtained from all participants.

### 2.2 Predictive factors

Based on Social Cognitive Theory, this study categorizes 26 predictive factors into three domains: environmental factors, personal factors, and behavioral responses. Environmental factors include: education level, monthly income, marital status, residence area, occupation, surgery status, chemotherapy status, and social support. Personal factors include: age, BMI, C-reactive protein, hemoglobin, HDL, LDL, triglycerides, fasting blood glucose, presence of comorbidities, hearing function, language function, anxiety, and depression. Behavioral responses include: smoking status, alcohol consumption, physical exercise, Sleep duration, and medication adherence.

### 2.3 Sample size

Although no universal approach exists for determining sample size in surveys utilizing machine learning methods, LAP generally suggests an initial sample of 500 or more (30). Moreover, the sample size can be determined using the Events Per Variable (EPV) approach (31), which recommends having a minimum of 10 events per predictor or outcome variable to guarantee reliable and stable results. This research incorporates 26 predictive elements, necessitating at least 260 subjects. With a total of 666 participants enrolled, the study satisfied both analytical requirements, thereby bolstering the reliability and clinical relevance of the outcomes.

### 2.4 Measure

#### 2.4.1 General information questionnaire

The General Information Questionnaire includes two parts: demographic details and illness-related factors. The demographic section includes age, marital status, monthly income, education level, residence area, medication adherence, and sleep duration, etc. The disease-related section includes factors such as whether the patient has undergone surgery or chemotherapy.

#### 2.4.2 Benefit finding scale

The BFS was initially created by Antoni et al. to assess benefit finding among women with early-stage breast cancer (32). The Chinese version of the BFS was adapted and revised by Liu Zhunjun et al., consisting of six dimensions with 22 items: Acceptance, Family Relationships, Personal Growth, Worldview, Social Relationships, and Health (15). The scale boasts a Cronbach’s α of 0.95 and a validity score of 0.97, demonstrating strong reliability and accuracy. It employs a 5-point Likert scale, with responses ranging from 1 (“Not at all”) to 5 (“Very much”), capturing varying degrees of agreement or intensity. The cumulative score, derived by adding the scores across all dimensions, falls within a range of 22 to 110. Higher totals signify a greater ability to find benefits in challenging situations, whereas lower scores suggest a diminished capacity for such insight. In this particular study, the Chinese adaptation of the Benefit Finding Scale achieved a Cronbach’s α of 0.834.

#### 2.4.3 Hospital anxiety and depression scale

The HADS stands out as a go-to instrument for assessing anxiety and depression, featuring a 14-item structure split into two distinct categories: Anxiety and Depression. Scores on this scale span from 0 to 21, with a threshold set at 8. Falling below this mark typically signals an absence of symptoms, whereas hitting 8 or higher points to the likelihood of their presence (33). The Chinese adaptation of the HADS has proven to be both reliable and valid when used with cancer patients, boasting an impressive Cronbach’s α of 0.929 for the overall scale. Additionally, the Anxiety and Depression subscales each scored a solid 0.874, further underscoring the tool’s robustness in this context (34). In this research, the HADS Cronbach’s α value was 0.802.

#### 2.4.4 Social support rating scale

The SSRS, created by Xiao Shuiyuan in 1986 and updated in 1990, measures social support, particularly among older adults (35). The assessment tool comprises three key aspects: Objective Support, Subjective Support, and Support Utilization, featuring 10 items and a top score of 66. Scoring below 22 suggests minimal social support, while a range of 22 to 44 points to moderate support. Scores exceeding 44 signify robust social support, with higher values indicating stronger support systems. In this study, the scale demonstrated strong reliability, with a Cronbach’s α coefficient of 0.845.

### 2.5 Data collection methods and quality control

The research team was composed of master’s students and clinical research nurses, all of whom underwent standardized training. Upon obtaining approval from the relevant hospital departments, the researchers employed a standardized script to guide eligible breast cancer patients through the process of completing the questionnaires. The researchers thoroughly explained the study’s objectives, significance, and instructions for completing the questionnaires. Following the acquisition of informed consent from the participants, the questionnaires were distributed. Any queries raised by the participants during the completion of the forms were addressed immediately on-site. Upon completion, the questionnaires were collected without delay, and those containing incorrect responses were excluded from the analysis.

To ensure scientific rigor and efficiency, the data collection process adhered to strict operational protocols. The procedures for obtaining informed consent, providing on-site guidance, and collecting completed questionnaires were carefully controlled to maintain high-quality standards. A total of 711 questionnaires were distributed, with 666 valid responses collected, yielding an effective response rate of 93.6%. These data provide a reliable foundation for subsequent analysis.

### 2.6 Handling of missing data

In this study, all questionnaires underwent completeness checks after collection. Any questionnaire with missing items was deemed invalid and excluded prior to data analysis. This approach ensured that the final 666 questionnaires included in the analysis were complete and contained no missing values. This method is commonly employed in cross-sectional studies to guarantee data consistency and the validity of analyses.

### 2.7 Statistical analysis

Latent Profile analysis was conducted using Mplus 8.3 software to investigate the underlying characteristics of benefits identified in breast cancer patients. The analysis commenced with a unidimensional model, progressively incorporating additional categories until the model fit indices were optimized. The information criteria encompass the Akaike Information Criterion (AIC), Bayesian Information Criterion (BIC), and the adjusted Bayesian Information Criterion (aBIC), where lower values signify a superior model fit. The entropy index spans from 0 to 1 and serves as a measure of classification accuracy, where values exceeding 0.8 suggest that accuracy surpasses 90% (36). Likelihood ratio test statistics, including the Lo-Mendell-Rubin Test (LMRT) and the Bootstrap Likelihood Ratio Test (BLRT), are employed to compare alternative latent class models, with a P-value of less than 0.05 signifying that the k-class model outperforms the k-1 class model (37). The best classification model was chosen in accordance with these integrated criteria.

This study summarized participants’ general characteristics using descriptive statistics. Continuous variables not following normal distribution were presented as median and interquartile range, while categorical variables were expressed as counts and percentages. Univariate analysis employed the Mann-Whitney U test, chi-square test, or Fisher’s exact test. Univariate analysis was performed using R 4.5.2, with a two-tailed significance threshold of *P* < 0.05. After determining the optimal model and classification, we assessed differences in environmental factors, personal factors, and behavioral responses among benefit finding groups categorized by different classifications. Important variables from univariate analysis (p ≤ 0.05) were further analyzed using the Least Absolute Shrinkage and Selection Operator (LASSO) regression to retain significant predictors. Then, binary logistic regression is conducted to determine the influencing factors of benefit finding. Five machine learning algorithms—Logistic Regression, Decision Tree, XGBoost, SVM, and ANN—were employed to build and compare benefit finding prediction models. All models were implemented using Python 3.13. Additionally, model training employed bootstrap resampling. Performance was evaluated using metrics including area under the receiver operating characteristic curve (ROC), accuracy, sensitivity, specificity, positive predictive value, negative predictive value, and F1 score. Calibration was assessed by comparing predicted versus observed “low benefit finding category” incidence rates. Model discriminative capability was evaluated via ROC analysis, quantified by AUC. Calibration plots assessed consistency between predicted probabilities and actual outcomes. Decision curve analysis (DCA) evaluated clinical utility and net benefit. Feature importance was assessed using Shapley additive explanations (SHAP) analysis, where higher SHAP absolute values indicate greater influence on predictions. Additionally, we explored the distribution of feature values and their relationship with model predictions to further understand model behavior. SHAP analysis was also performed using Python 3.13.

## 3 Results

### 3.1 General data analysis of breast cancer patients

This study included 666 breast cancer patients, with demographic classification showing 343 individuals (51.50%) aged under 60. Regarding marital status, the majority (specifically 92.64%) were married, while a minority (7.36%) were unmarried, divorced, or widowed. Among participants, 281 individuals (42.19%) had a monthly income below 2,000 RMB. Additionally, 346 individuals (51.95%) resided in rural areas, 215 (32.28%) were illiterate, 440 (66.07%) underwent breast cancer surgery, and 331 (49.7%) received chemotherapy. Detailed patient information is presented in **Table 1**.

**Table 1:** Univariate analysis of the potential categories of benefit finding in breast cancer patients [example (%)].

Variable	Overall	Category 1	Category 2	Statistics	*P*
Age				158.99^b^	<0.001
<60 years old	343 (51.50)	42 (18.10)	301 (69.35)		
≥60 years old	323 (48.50)	190 (81.90)	133 (30.65)		
Marital status				2.49^b^	0.114
Unmarried	49 (7.36)	12 (5.17)	37 (8.53)		
Married	617 (92.64)	220 (94.83)	397 (91.47)		
Monthly income				4.61^b^	0.1
≤2000CNY	281 (42.19)	108 (46.55)	173 (39.86)		
2000–5000 CNY	245 (36.79)	85 (36.64)	160 (36.87)		
≥5000 CNY	140 (21.02)	39 (16.81)	101 (23.27)		
Education level				1.02^b^	0.6
Illiterate	215 (32.28)	79 (34.05)	136 (31.34)		
0–6 Years	187 (28.08)	67 (28.88)	120 (27.65)		
> 6 Years	264 (39.64)	86 (37.07)	178 (41.01)		
Residence area				1.50	0.220
Rural	346 (51.95)	113 (48.71)	233 (53.69)		
Urban	320 (48.05)	119 (51.29)	201 (46.31)		
Smoking status				5.46^b^	0.065
Smoker	78 (11.71)	21 (9.05)	57 (13.13)		
Ex-Smoker	102 (15.32)	29 (12.50)	73 (16.82)		
Non-smoker	486 (72.97)	182 (78.45)	304 (70.05)		
Alcohol consumption				1.37^b^	0.503
Non-drinker	494 (74.17)	177 (76.29)	317 (73.04)		
Light drinker	112 (16.82)	38 (16.38)	74 (17.05)		
Heavy drinker	60 (9.01)	17 (7.33)	43 (9.91)		
Physical exercise				0.01^b^	0.927
Exercise ≥ once per week	392 (58.86)	136 (58.62)	256 (58.99)		
No exercise	274 (41.14)	96 (41.38)	178 (41.01)		
Occupation				1.26^b^	0.261
Low-skill work	467 (70.12)	169 (72.84)	298 (68.66)		
Knowledge-based work	199 (29.88)	63 (27.16)	136 (31.34)		
Sleep duration				2.04^b^	0.153
≥ 6 hours	341 (51.20)	110 (47.41)	231 (53.23)		
< 6 hours	325 (48.80)	122 (52.59)	203 (46.77)		
Surgery status				0.02^b^	0.901
Had surgery	440 (66.07)	154 (66.38)	286 (65.90)		
No surgery	226 (33.93)	78 (33.62)	148 (34.10)		
Chemotherapy status				1.57^b^	0.211
No Chemotherapy	331 (49.70)	123 (53.02)	208 (47.93)		
Had Chemotherapy	335 (50.30)	109 (46.98)	226 (52.07)		
Presence of comorbidities				0.93^b^	0.335
Yes	242 (36.34)	90 (38.79)	152 (35.02)		
No	424 (63.66)	142 (61.21)	282 (64.98)		
Hearing function				0.02^b^	0.882
Normal hearing	593 (89.04)	206 (88.79)	387 (89.17)		
Hearing loss	73 (10.96)	26 (11.21)	47 (10.83)		
Language function				1.64^b^	0.201
Normal	647 (97.15)	228 (98.28)	419 (96.54)		
Abnormal	19 (2.85)	4 (1.72)	15 (3.46)		
Medication adherence				159.73^b^	<0.001
Yes	468 (70.27)	92 (39.66)	376 (86.64)		
No	198 (29.73)	140 (60.34)	58 (13.36)		
C-reactive protein	5.00 [5.00, 5.00]	5.00 [5.00, 5.00]	5.00 [5.00, 5.00]	-0.02^a^	0.985
Hemoglobin	122.00 [114.00, 131.00]	124.00 [116.00, 133.00]	122.00 [113.00, 131.00]	-1.38^a^	0.168
HDL	1.41 [1.22, 1.70]	1.38 [1.22, 1.67]	1.42 [1.24, 1.70]	-1.78^a^	0.076
LDL	2.88 [2.14, 3.30]	3.03 [2.17, 3.39]	2.86 [2.09, 3.30]	-1.54^a^	0.124
Triglycerides	1.71 [1.19, 2.39]	1.62 [1.09, 2.26]	1.71 [1.23, 2.47]	-1.39^a^	0.164
Fasting blood glucose	5.23 [4.98, 6.03]	5.23 [4.98, 6.03]	5.23 [4.98, 6.03]	-0.20^a^	0.841
BMI	25.91 [23.05, 28.84]	25.71 [23.14, 28.36]	25.96 [23.00, 28.84]	-0.10^a^	0.918
SSRS	32.00 [24.00, 38.00]	25.00 [19.00, 37.00]	33.00 [28.00, 39.00]	-7.01^a^	<0.001
HADS-A	8.00 [5.00, 14.00]	13.50 [8.00, 16.00]	7.00 [4.00, 10.00]	-11.24^a^	<0.001
HADS-D	7.00 [4.00, 10.00]	9.00 [5.00, 13.00]	6.00 [3.00, 9.00]	-6.03^a^	<0.001

The Hospital Anxiety and Depression Scale is divided into two components: HADS-A, which specifically measures anxiety, and HADS-D, which focuses on depression. Additionally, the Social Support Rating Scale, abbreviated as SSRS, is used to assess the level of social support individuals receive. Category 1, Low Benefit Finding Category; Category 2, High Benefit Finding Category; a, Mann-Whitney test; b, Chi-square test.

### 3.2 Latent profile analysis of benefit finding in breast cancer patients

This study is based on latent feature analysis. By comparing the fit indices of Models 1 to 4, Model 2 was ultimately selected as the optimal model. The rationale for this decision includes: information criteria (AIC, BIC, aBIC) showed a consistent downward trend; high entropy (0.970) indicated good classification accuracy; and likelihood ratio tests (LMR-LRT and BLRT) demonstrated that the two-class model significantly outperformed the one-class model. Furthermore, the two-category model demonstrated balanced category distribution and theoretical interpretability, aligning with the research objectives and facilitating in-depth analysis of subsequent influencing factors. In contrast, the three- and four-category models exhibited categories with extremely low proportions (e.g., 3%), potentially representing outliers or random variation within the sample rather than universally significant latent subgroups. The stability and reproducibility of these categories are questionable. Therefore, I selected the binary classification as the optimal model. **Table 2** displays the model fit indices for models ranging from 1 to 4 categories.

**Table 2:** Displays the latent profile analysis of advantages recognized in breast cancer patients.

Model	AIC	BIC	aBIC	Entropy	*P*		Probability of classes
					*LMR-LRT*	*BLRT*	
1	18678.114	18732.129	18694.029				
2	17612.104	17697.629	17637.302	0.970	0.000	0.000	0.35/0.65
3	17352.200	17469.234	17386.682	0.979	0.0030	0.000	0.35/0.62/0.03
4	17233.729	17382.272	17277.495	0.980	0.0022	0.000	0.35/0.59/0.03/0.03

### 3.3 The nomenclature of potential subtypes associated with the benefit finding in breast cancer patients

**Figure 1:**
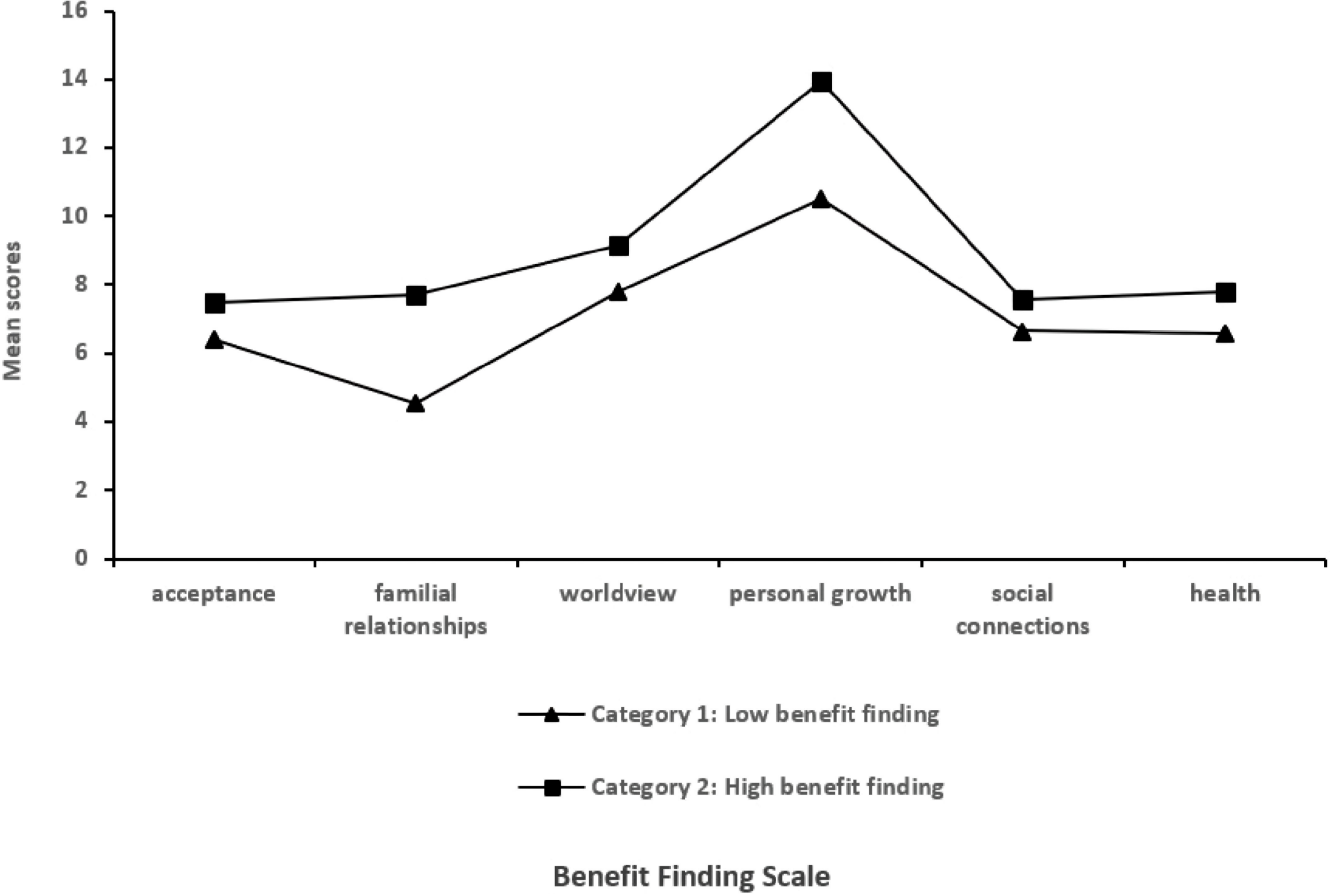
The distribution of features of potential categories associated with the benefits in breast cancer patients was identified.

Based on the scoring characteristics of each category, this study found that the average score for Category 1 was 42.47 ± 5.74 points. The mean scores across all dimensions were the lowest among all categories. Therefore, Category 1 was designated as the low benefit finding category (35%). Category 2 had a mean score of (59.57 ± 7.09), with the highest mean scores across all dimensions among all categories. This category was named the high benefit finding category (65%). The mean scores for each dimension of the Breast Cancer Patient Benefit Assessment Scale are shown in **Figure 1**.

### 3.4 Univariate analysis and LASSO regression of potential determinants of benefit finding

**Figure 2:**
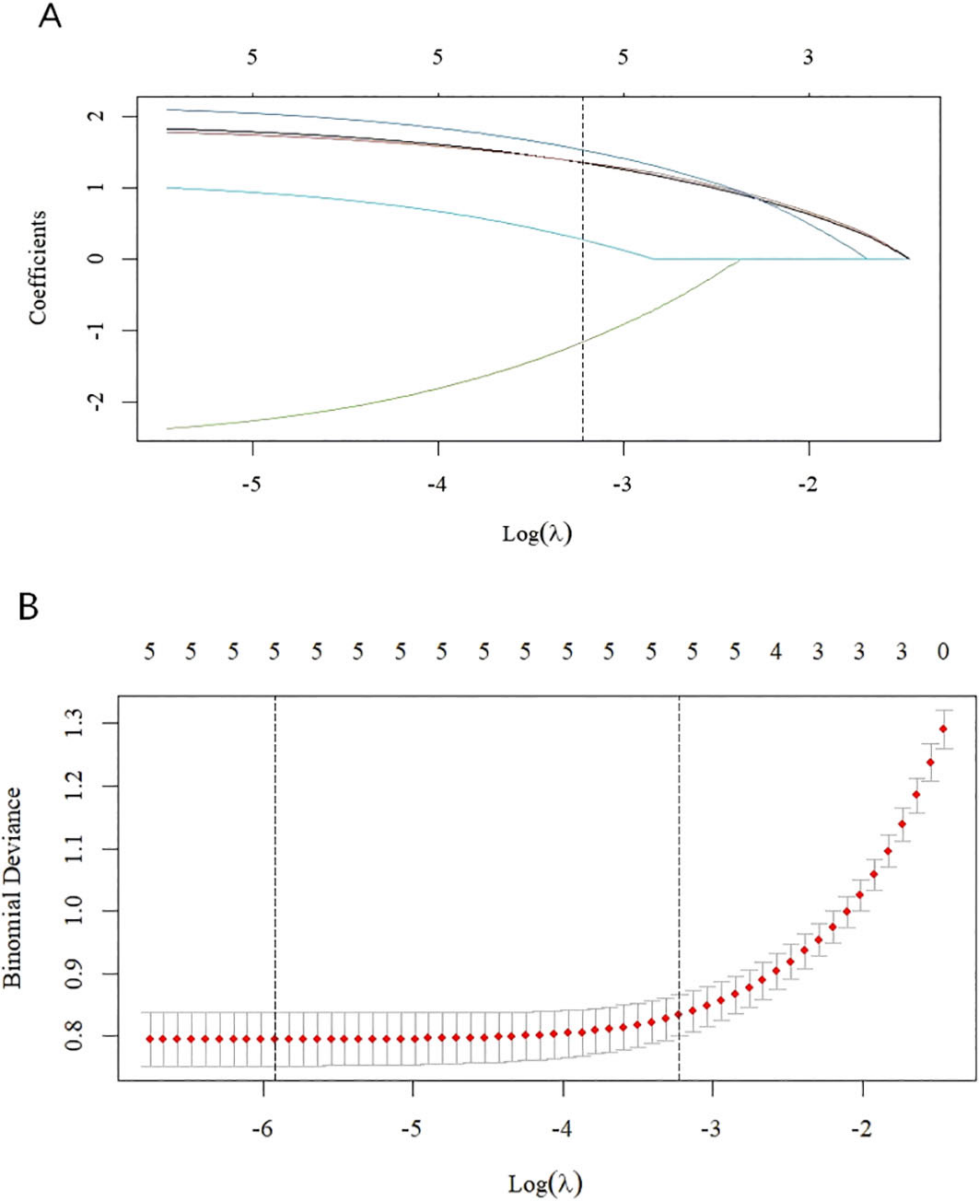
Feature selection based on LASSO regression. Coefficient distribution plot **(A)** and penalty plot **(B)**. The optimal value for the dashed vertical line is selected as the minimum standard deviation and one standard deviation above the minimum standard deviation.

Univariate analysis revealed statistically significant differences (P < 0.05) between benefit finding categories across the following variables: age, medication adherence, anxiety, depression, and social support. **Table 1** presents the results of the univariate analysis. LASSO regression indicated that age, medication adherence, anxiety, depression, and social support were statistically significant (see **Figure 2**).

### 3.5 Binary logistic regression analysis

Using the high benefit finding category (C2) as a reference, binary logistic regression analysis was employed to identify key factors influencing benefit classification. Anxiety (OR = 1.116, P < 0.001) and depression (OR = 1.064, P = 0.01) were associated with a higher likelihood of belonging to the low benefit finding category. Age < 60 years (OR = 0.148, P < 0.001), good medication adherence (OR = 0.156, P < 0.001), and social support (OR = 0.95, P = 0.01) were more likely to be associated with the high benefit finding category. The comprehensive results of the binary logistic regression are shown in **Table 3**.

**Table 3:** Binary logistic regression examination of benefit identification in breast cancer patients.

Variable	β	*SE*	Waldχ^2^	*OR*	95%CI	*P*
(Intercept)	1.523	0.556	2.739	4.585	1.542-13.63	0.006
Age1	-1.911	0.234	-8.17	0.148	0.094-0.234	<0.001
Medication Adherence1	-1.857	0.247	-7.522	0.156	0.096-0.253	<0.001
Social Support	-0.051	0.012	-4.454	0.95	0.929-0.972	<0.001
Anxiety	0.11	0.021	5.239	1.116	1.071-1.162	<0.001
Depression	0.062	0.024	2.587	1.064	1.015-1.116	0.01

Age1:<60 years old, Medication Adherence1: Good medication adherence.

### 3.6 Comparative analysis and optimization of multiple machine learning models

**Figure 3:**
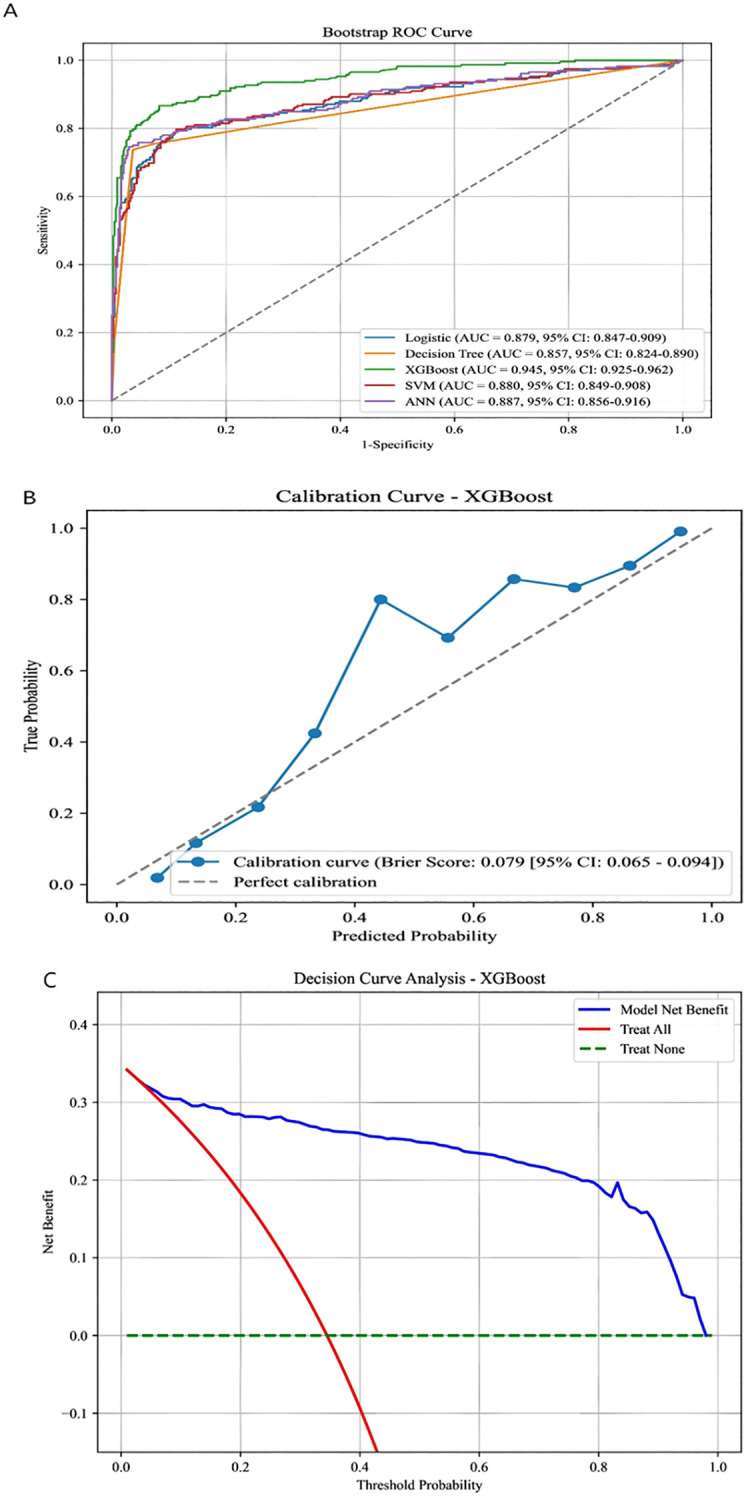
**(A)** Comparison of ROC curves for five machine learning algorithms predicting low benefit finding in Bootstrap. **(B)** Calibration curve for predicting low benefit finding. **(C)** Decision curve for predicting low benefit finding.

We evaluated five machine learning models for predicting patient benefit finding in breast cancer. The XGBoost model achieved an AUC value of 0.945 and demonstrated significantly superior sensitivity and specificity compared to the other four models, thereby being selected as the final model. Results are shown in **Figure 3A**. The calibration curve in **Figure 3B** demonstrates a close correspondence between predicted probabilities and actual outcomes. Furthermore, the decision curve analysis in **Figure 3C** unequivocally highlights this model’s superior predictive capability compared to others, offering substantial benefits to relevant patients.

### 3.7 Model interpretation

**Figure 4:**
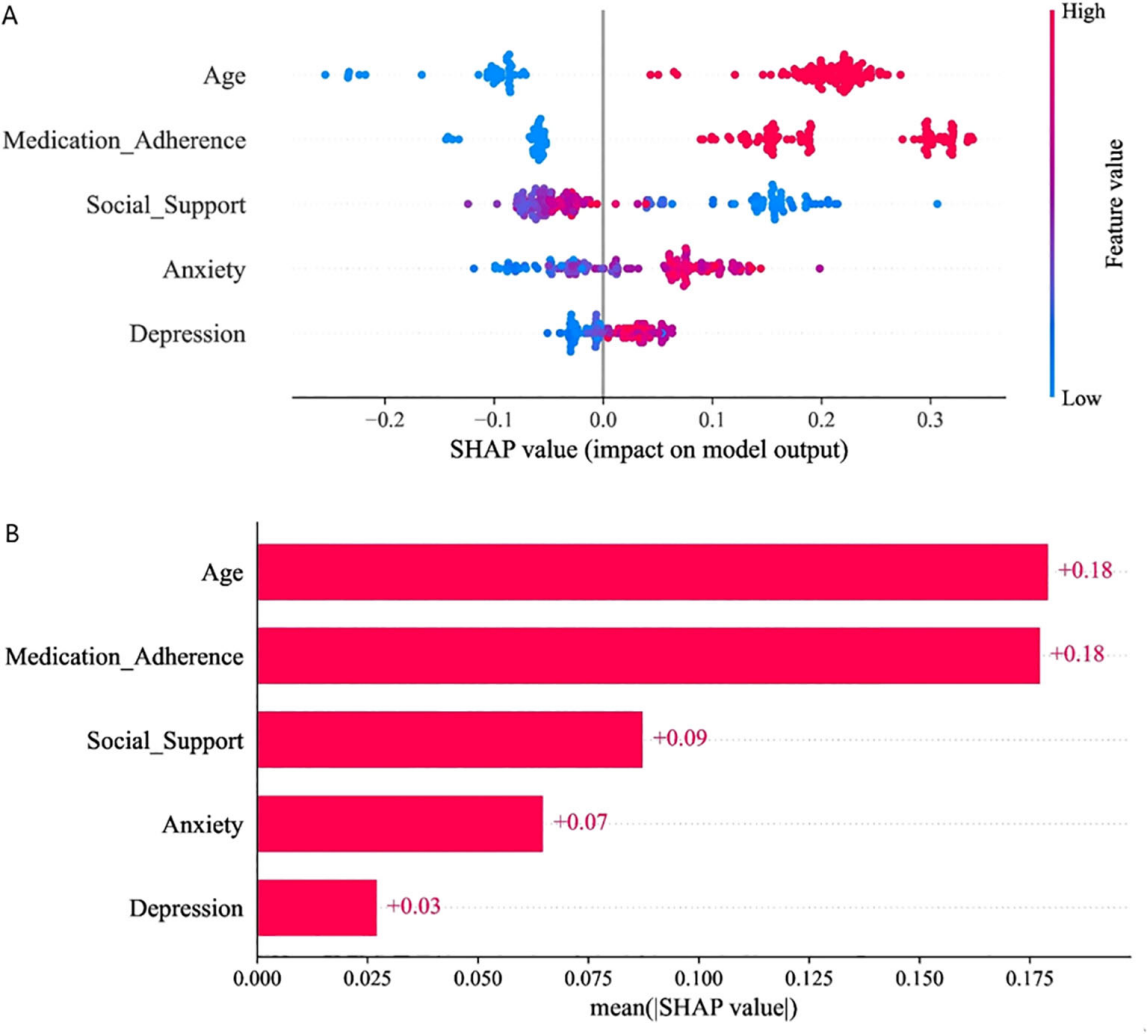
Interpretation of XGBoost model results based on the SHAP method. SHAP summary plot **(A)** and bar chart **(B)**.

**Figure 5:**
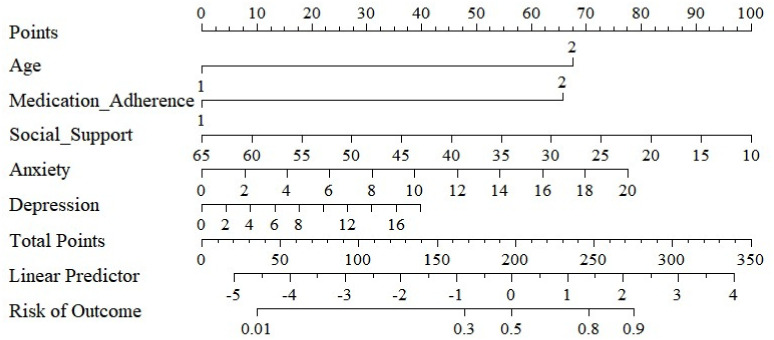
Nomogram predicting the probability of low benefit finding in breast cancer patients.

**Figure 4** illustrates the impact of various features on model prediction outcomes. As shown in **Figure 4A**, the SHAP summary plot ranks the importance of five key features. The average absolute SHAP value histogram (**Figure 4B**) further corroborates this finding. These data identify which feature values significantly influence the final risk prediction score and cause its fluctuations. Additionally, this study developed a nomogram, with results presented in **Figure 5**.

## 4 Discussion

### 4.1 Latent profile analysis of benefits identified for breast cancer patients

The BFS score of 666 breast cancer patients in this study was (53.62 ± 10.52), which was significantly lower than the values reported in domestic studies (38, 39), and this might be related to multiple factors. The negative impact of mastectomy on body image and self-esteem may be amplified in Chinese culture due to the emphasis on bodily integrity, leading to more pronounced psychological distress (40). Additionally, side effects from treatments like chemotherapy may also contribute to lower BFS scores (41).

Through latent profile analysis, this study identified significant heterogeneity in benefit finding among breast cancer patients, categorizing them into two distinct categories: the low benefit finding category (35%) and the high benefit finding category (65%). The low benefit finding category scored significantly lower than the high benefit finding category on the “family relationships” dimension. This finding suggests that breast cancer patients who struggle to perceive positive meaning from their illness tend to experience higher levels of negative emotions. This, in turn, diminishes their willingness to engage with family members and reduces the frequency of emotional exchanges, ultimately leading to decreased family closeness and overall adaptive functioning (42). The high benefit finding category exhibited significantly higher BFS scores than the low benefit finding category, with particularly prominent scores in the personal growth dimension. This finding indicates that social support from family, friends, and healthcare teams not only provides emotional comfort and practical assistance to breast cancer patients, alleviating negative emotions triggered by the disease (such as anxiety and inferiority), but also reduces psychological burdens, prompting patients to re-examine their disease experiences. By uncovering positive meanings within the treatment process, interpersonal interactions, and personal growth, this support significantly enhances patients’ levels of benefit finding (43). Breast cancer patients exhibit individual differences in benefit finding. Therefore, healthcare providers need to tailor their care approaches to the specific needs of each patient group and implement targeted interventions.

### 4.2 Personal factors include: Age, anxiety, and depression

The results of this study indicate that breast cancer patients under the age of 60 are more likely to be classified as a high benefit finding category. Some studies have shown that younger patients are more likely to achieve positive cognitive outcomes from their disease experiences, such as personal growth and relationship enhancement (44, 45). However, other studies indicate a positive correlation between age and benefit finding, suggesting that older patients may exhibit greater psychological growth across various dimensions (e.g., in “acceptance” and “life perspective”) due to accumulated life experiences, mature coping strategies, and meaning-making processes (46). This inconsistency may arise from variations in cultural contexts and assessment tools. In Asian collectivist cultures, older patients often prioritize family harmony and role adaptation (47), resulting in distinct expressions of benefit finding compared to Western individualistic cultures that emphasize personal growth (48). From a theoretical perspective, the meaning-making process within social cognitive theory may explain age differences: younger patients, being more future-oriented, tend to view illness as a life transition point, thereby stimulating growth motivation (49); whereas older individuals may rely more heavily on existing coping resources and life narratives to integrate illness experiences into their life stories. Additionally, older patients often face multiple health issues and diminished social support, which may influence their ability to discover benefits (50, 51). For younger patients, cognitive restructuring training guided by oncology nurses or psychologists can be initiated during the initial diagnosis and adjuvant therapy phases to enhance their understanding of treatment significance. Peer support groups can also be utilized to facilitate experience sharing (52, 53). For elderly patients, maintaining their social support systems is paramount. An interdisciplinary team comprising social workers and geriatric care specialists should assist these patients in managing their disease while preserving or restoring quality of life, thereby enhancing benefit finding (54). Additionally, mindfulness-based stress reduction therapy can alleviate anxiety and fatigue during and after chemotherapy across all age groups, indirectly promoting benefit finding (55).

This study confirms that anxiety is more readily categorized under the low benefit finding category. This finding aligns strongly with current mainstream theoretical frameworks and empirical research evidence. From the perspective of cognitive adaptation theory (56), individuals facing major threats such as cancer achieve psychological adaptation through meaning reconstruction, regaining a sense of control, and self-enhancement. Benefit finding is the core manifestation of this adaptive cognitive process (56, 57). High levels of anxiety, as an emotional state that continuously depletes psychological resources, severely impede patients’ ability to initiate and sustain this positive cognitive reappraisal. It causes their cognitive focus to become fixated on the disease’s threats and uncertainties, making it difficult to uncover positive meanings such as personal growth, improved relationships, or shifts in life philosophy from their experiences (58). Moreover, under high anxiety, patients tend to focus more on the threatening aspects of illness (e.g., recurrence risk, treatment side effects) rather than potential positive changes (e.g., improved family relationships, personal growth). This leads to cognitive processing biased toward negative information, inhibiting the emergence of benefit finding (21). Therefore, this study proposes a clinical practice framework: stepwise psychosocial interventions should be systematically integrated into routine breast cancer treatment. Regarding intervention types, evidence-based approaches such as Mindfulness-Based Stress Reduction (MBSR) and Acceptance and Commitment Therapy (ACT) should be prioritized to manage anxiety and cultivate meaning finding (59). These may be supplemented with group interventions centered on benefit finding, such as narrative therapy (60). At critical stages (e.g., initial diagnosis and adjuvant therapy), stepwise psychological care should be implemented (61). The stepped care model builds upon routine care through four progressive stages: observation, guidance, face-to-face guidance, and professional intervention. Through tiered interventions including health education, WeChat group guidance, follow-up practical training, and professional psychological counseling, it effectively alleviates psychological distress and anxiety in breast cancer patients undergoing postoperative chemotherapy while significantly improving their quality of life. This approach aims to proactively promote psychological growth and long-term adaptation by specifically alleviating anxiety, ultimately enhancing the level of perceived benefits.

Depression is associated with the category of low benefit finding. According to the stress generation hypothesis (62), depressive symptoms can lead individuals to develop negative thoughts, emotions, and behaviors, which may negatively affect body image and have adverse impacts on health outcomes for cancer patients (63). This, in turn, hinders their ability to find benefit (64). Across both domestic and international contexts, studies consistently report an inverse association between depression and BF levels (18, 21, 65). Thus, the outcomes of our investigation align nicely with what has been previously documented. From a clinical perspective, these results have practical implications. First, early identification and intervention of depressive symptoms during cancer rehabilitation are crucial to prevent their progression and avoid negative effects on the psychological recovery of patients. Secondly, CBT and mindfulness-based interventions not only alleviate depressive symptoms but also enhance emotional regulation. By doing so, these methods boost psychological well-being, elevate life satisfaction, and build greater mental resilience.

### 4.3 Behavioral response: medication adherence

This study indicates that patients with good medication adherence tend to fall into the high benefit finding category. This finding aligns with research by Neugut et al., which shows that patients completing adjuvant chemotherapy regimens often report new perspectives on life and greater perceived treatment benefits (66). From the perspective of self-determination theory, good adherence reflects not only behavioral compliance but also likely indicates patients’ internal identification with and meaning integration of treatment (67). This intrinsic motivation helps reinforce patients’ sense of “self-efficacy,” thereby reshaping their perception of the disease and enhancing benefit finding (68). Medication adherence enhances patients’ sense of control over their illness, improves self-management capabilities, and facilitates proactive coping strategies, thereby increasing benefit finding (69). Furthermore, research indicates that positive psychological factors and social support significantly promote long-term adherence (70, 71), with such support itself serving as a crucial catalyst for benefit finding. Therefore, clinical interventions should integrate adherence management with psychosocial support. For instance, incorporating structured adherence counseling led by nurses or clinical pharmacists trained in motivational interviewing during routine oncology follow-ups can help patients connect medication adherence with overall recovery goals (72). Concurrently, family-centered positive psychological interventions effectively enhance psychological resilience, hope, perceived disease benefits, and quality of life in breast cancer patients (73).

### 4.4 Environmental factors: social support

Social support is associated with a high benefit finding category. The study revealed that social support enhances benefit finding both directly and indirectly by boosting patients’ hope (74). Sufficient social support can increase cancer patients’ hope (75), help them maintain an optimistic attitude toward life, and reduce the suffering caused by physical pain and unpleasant experiences, ultimately leading them to find meaning in life and enhance their BF levels (76). Additionally, other studies have demonstrated a positive link between social support and BF (20). Therefore, strengthening social support to improve BF levels is particularly important. Medical teams can provide resource navigation (such as electronic social resource lists), conduct family empowerment training (communication skills/symptom management), and strengthen both instrumental and emotional support to enhance patients’ BF.

### 4.5 Clinical implications

This study combines social cognitive theory, latent profile analysis, and machine learning to reveal, for the first time, significant heterogeneity in perceived benefits among breast cancer patients, identifying a “low benefit finding category” (35%) and a “high benefit finding category” (65%). It further identifies age, medication adherence, social support, anxiety, and depression as key influencing factors. Based on these findings, clinical practice should implement individualized interventions. For high-risk patients characterized by advanced age, low social support, high anxiety/depression, and poor medication adherence, healthcare teams can utilize the developed predictive model for early identification. Comprehensive measures should be implemented, including stepwise psychological interventions (e.g., CBT, mindfulness therapy), structured adherence counseling, and reinforcement of family social support. These approaches proactively promote psychological adaptation and meaning-making, ultimately enhancing overall psychological recovery and quality of life.

### 4.6 Study limitations

This research is not without its shortcomings. For starters, the cross-sectional approach falls short of tracking the evolving advantages for breast cancer patients as time progresses. Additionally, the fact that all subjects were drawn exclusively from Anhui Province raises questions about how well these results can be extrapolated to different areas or demographic groups. Additionally, the questionnaires in this study were voluntarily completed by breast cancer patients. Despite excluding non-compliant responses and addressing missing data during quality control, there remains a potential for response bias. Therefore, future studies could improve upon this research by increasing the sample size, enhancing regional diversity, and employing longitudinal and qualitative methods to further explore the factors influencing breast cancer patients’ perceptions of benefits.

## 5 Conclusion

This study represents the first attempt to integrate the social cognitive theory framework with latent profile analysis (LPA) and modern machine learning methods to develop a predictive model for breast cancer patient benefit finding. Through LPA, we identified two distinct benefit finding subgroups exhibiting significant heterogeneity, revealing different psychological adaptation patterns among patients. The XGBoost model demonstrated exceptional predictive capability (AUC = 0.945), while SHAP interpretability analysis highlighted five key influencing factors: age, medication adherence, social support, anxiety, and depression. This underscores the complex interplay of environmental, personal, and behavioral factors affecting benefit finding. The nomogram we developed provides healthcare professionals with a practical, rapid method to identify patients with low benefit finding for timely psychological interventions such as cognitive behavioral therapy, mindfulness training, and enhanced social support. This will facilitate personalized care strategies to mitigate adverse mental health outcomes and improve patients’ long-term quality of life.

## Data Availability

The raw data supporting the conclusions of this article will be made available by the authors, without undue reservation.
